# Experimental Studies of Thermophysical Properties and Microstructure of X37CrMoV5-1 Hot-Work Tool Steel and Maraging 350 Steel

**DOI:** 10.3390/ma16031206

**Published:** 2023-01-31

**Authors:** Piotr Koniorczyk, Mateusz Zieliński, Judyta Sienkiewicz, Janusz Zmywaczyk, Andrzej Dębski

**Affiliations:** Faculty of Mechatronics, Armament and Aerospace, Military University of Technology, Ul. Gen. S. Kaliskiego 2, 00-908 Warsaw, Poland

**Keywords:** X37CrMoV5-1 (1.2343) steel, maraging 350 (1.6355) steel, microstructures, thermal diffusivity, specific heat capacity, thermal expansion

## Abstract

Measurements of thermal diffusivity, heat capacity and thermal expansion of X37CrMoV5-1 (1.2343) hot-work tool steel and Maraging 350 (1.6355) steel in the temperature range from −50 °C to 1000 °C were carried out in this paper. Both X37CrMoV5-1 and Maraging 350 are tested for military use as barrel steels. Thermophysical properties were tested using specialised test stands from NETZSCH. Thermal diffusivity was studied using both the LFA 427 laser flash apparatus in the temperature range of RT–1000 °C and the LFA 467 laser flash apparatus in the temperature range of −50 °C–500 °C. Specific heat capacity was investigated using a DSC 404 F1 Pegasus differential scanning calorimeter in the range RT–1000 °C, and thermal expansion was investigated using both a DIL 402 Expedis pushrod dilatometer in the range −50 °C–500 °C and a DIL 402 C in the range RT–1000 °C. Inconel 600 was selected as the reference material during the thermal diffusivity test using LFA467. Tests under the light microscope (LM), scanning electron microscopy (SEM) and Vickers microhardness measurements were carried out to detect changes in the microstructure before and after thermophysical measurements. This paper briefly characterises the research procedures used. In conclusion, the results of testing the thermophysical properties of X37CrMoV5-1 hot-work tool steel and Maraging 350 steel are compared with our results on 38HMJ (1.8509), 30HN2MFA and Duplex (1.4462) barrel steels. The thermophysical properties of X37CrMoV5-1 (1.2343) hot-work tool steel and Maraging 350 (1.6355) steel are incomplete in the literature. The paper presents the thermophysical properties of these steels over a wide range of temperatures so that they can be used as input data for numerical simulations of heat transfer in cannon barrels.

## 1. Introduction

While operating anti-aircraft guns, the destruction of the inner surface of the barrel caused by overheating is frequently observed. Such deterioration is manifested by the network of cracks and degradation of the protective chromium coating [1,2,3,4,5,6]. The temperature of the inner surface of the barrel of such weapons during a series of shots exceeds 1100 °C [7,8,9]. This is indicated by the results of numerical simulations of heat transfer and experimental tests, which showed the presence of molten copper in the gaps under the galvanic layer of chromium [2]. Thermal loads in the barrel lead to structural changes, which then cause a change in mechanical properties. Those structural changes are related to the ferrite–austenite phase transition at a temperature above 730 °C. As it is known, such a transition during the heating of steel is associated with an abrupt volume change. In the abovementioned case, the shrinkage caused the phase transition from the BCC to FCC crystallographic lattice [4,10,11,12]. As the shots are cycled, the cracks appearing in the barrel increase in size. After each shot, the barrel is thereafter cooled, and the austenite is turned back into ferrite. Moreover, high pressure shifts the austenite–ferrite transformation to a lower temperature. Altogether, the combined action of temperature and pressure causes deeper cracks to form and spread in the barrel and, as a consequence, it leads to the destruction of the chromium coating. In the first stage, the first cracks appear, followed by the formation of a network of cracks, and in the final stage, the coating falls away from the substrate. It is worth noting that even if the structural transformation covers a small cross-sectional area of the small-calibre gun barrel wall, it reduces the strength of the section. Also important is the cooling rate at which the nature of the transformation of austenite to ferrite and perlite changes depending on the time–temperature–precipitation (TTP) characteristics. The barrels of anti-aircraft weapons are characterised by a greater mass than the barrels of rifles and hence the slower cooling rate of the material. Earlier tests of the temperature characteristics of the 30HN2MFA, 38HMJ (1.8509) and Duplex barrel steels have shown that for 38HMJ steel the ferrite–austenite phase transition occurs at a temperature of about 790 °C, and for 30HN2MFA steel at about 730 °C, while for Duplex steel, such a change does not occur [1,13]. Steels with a higher temperature of phase transformation seem to be prospective, i.e., 38HMJ steel is better than 30HN2MFA steel. Heating 38HMJ and 30HN2MFA steel to 900 °C, followed by slow cooling, leads to irreversible changes in the structure consisting of the ferrite and pearlite with a characteristic Widmanstätten phase. This structure of the steel possesses a lower impact strength than the original one [14].

Over the years, large research groups have conducted many tests of thermophysical properties, i.e., thermal diffusivity, thermal conductivity, specific heat and thermal expansion and microstructures of both sheets of steel, i.e., X37CrMoV5-1 hot-work tool steel and Maraging 350 steel [14,15,16,17,18,19,20]. These experiments are becoming more accurate and verifiable, but are often incomplete and random. Thus, in the case of Maraging 350 steel, thermal diffusivity, thermal conductivity, specific heat and thermal expansion in the temperature range from RT to about 800 °C were presented [17]. In turn, the results of thermal expansion measurements of this steel in the temperature range from 400 °C to 900 °C are presented in [18]. Studies of the kinetics precipitation in steel of similar grade, e.g., in Maraging 250 steel using the DSC method in the temperature range from 300 °C to 800 °C, were studied in [16]. Studies of the microstructure of the Maraging 350 steel using the XRD method and with the use of SEM are presented in [14]. In the case of the X37CrMoV5-1 hot-work tool steel, the method of elaboration of specific heat input data for use in numerical simulations of heat transfer is presented in [15]. The authors of this paper argue with this concept in the summary of this paper. The thermal stability of the X37Cr MoV-5 hot-work tool steel microstructure after heat treatment with isothermal quenching and tempering was investigated [19]. The correlation between heat treatment, microstructure and mechanical properties of the X37CrMoV5-1 hot-work tool steel was investigated [20]. This study shows that for this steel, there is a strong dependence of mechanical properties on the microstructure. The results of the literature search indicate the lack of comprehensiveness of the research on thermophysical properties. However, it is often important to obtain the thermophysical properties of these steels over a wide range of temperature, so that they can be used as input data for numerical heat transfer simulations, e.g., in cannon barrels.

This work aims to investigate the thermophysical properties of the X37CrMoV5-1 hot-work tool steel, in which the ferrite–austenite phase transition occurs, and the Maraging 350 steel, in which there are no sudden changes in the structure under the influence of temperature, and thus is insensitive to overheating. The thermophysical properties of X37CrMoV5-1 hot-work tool steel and Maraging 350 one will be used as input data for conducting a numerical analysis of heat transfer in the barrel wall of a 35 mm calibre cannon, analogous to the case of 38HMJ, 30HN2MFA and Duplex steels [7].

## 2. Materials and Methods

### 2.1. Materials

The samples of X37CrMoV5-1 hot-work tool steel (BOSTAL, Bydgoszcz, Poland) and Maraging 350 (KAMB Import-Export, Warsaw, Poland) steel bar supplied for testing were in the form of a cylinder with a diameter of 20 mm and a length of 200 mm. The nominal composition (wt.%) of an X37CrMoV5-1 hot-work tool steel and Maraging 350 steel are listed in Table 1 and Table 2, respectively. Hot-work tool steel X37CrMoV5-1 is highly alloyed steel, designed to work at elevated temperatures. Compared to 38HMJ and 30HN2MFA steels, it possesses a higher temperature of ferrite–austenite transformation, which is around 840 °C [2]. Maraging 350 is a low-carbon iron–nickel martensitic steel hardened by precipitations of intermetallic phases [14]. The X37CrMoV5-1 hot-work tool steel and Maraging 350 steel adopted for the tests were not subjected to heat treatment by the authors. 

### 2.2. The X37CrMoV5-1 Time–Temperature-Transformation Diagram

**Figure 1:**
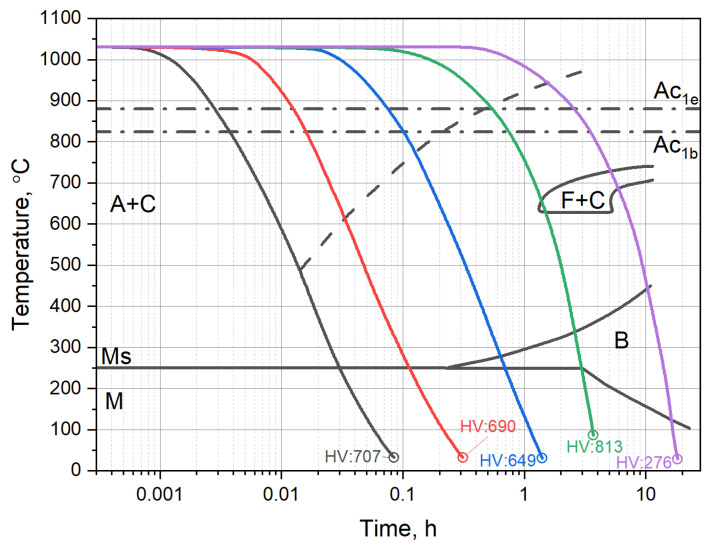
TTT curves (together with Continuous Cooling Transformation (CCT) curves) corresponding to the start of the precipitation of various phases in X37CrMoV5-1 hot-work tool steel; the letters stand for austenite (A), cementite (C), martensite (M), bainite (B), martensite start temperature (M_S_), ferrite (F) and austenite transformation temperature (Ac_1e_—start and Ac_1b_—end temperature of austenite transformation) [24].

Figure 1 shows the time–temperature-transformation curves for X37CrMoV5-1 hot-work tool steel. The Time–Temperature-Transformation (TTT) diagram demonstrates the expected microstructures according to the cooling path. Austenitic, bainitic and martensitic phase regions can be distinguished. Martensite starts at temperature M_S_ is constant at high cooling rates; while at lower cooling rates, it is dependent on the already transformed bainite. The formation of pearlite is shifted to long transformation times, which are not relevant for conventional heat treatment processes. 

The martensite phase formation is diffusionless, and hence martensite forms without any interchange in the position of neighbouring atoms. Martensite in steels is often plate-like or laths with a well-defined habit plane—the plane defined by the plate itself. Bainite transformation occurs like the martensitic one but with the partitioning of interstitial carbon. Bainite nucleates at a relatively high temperature wherein austenite is relatively weak and unable to elastically support large deformations [22,23]. As the transformation occurs at a higher temperature, bainite plates or lathes grow in a martensitic mechanism, and the excess carbon is quickly converted into residual austenite. At this point, the inter-plate carbides typical of an upper bainitic microstructure arise through the precipitation of carbon from austenite. As the transformation temperature is lowered, the post-transformation diffusion process slows down and with this, some of the carbon precipitates from supersaturated bainitic ferrite and the remainder partitions into the residual austenite, giving the classical lower bainitic microstructure. The pearlite is composed of layers of ferrite and cementite, formed by a eutectoid reaction from austenite. Pearlite is formed during sufficiently slow cooling.

### 2.3. The Maraging 350 Time–Temperature-Transformation Diagram

**Figure 2:**
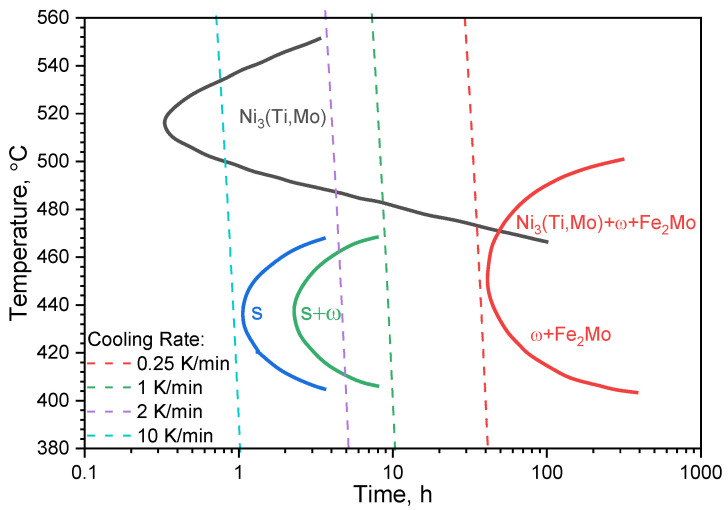
TTT curves correspond to the start of the precipitation of various phases in Maraging 350 steel (cooling rate was counted from 1000 °C) [14].

The schematic time–temperature-transformation curves (TTT) for the precipitation of various phases in Maraging steel are shown in Figure 2. The TTT diagram shows that all precipitates arise above 400 °C. As it is seen, at the temperature above 450 °C, precipitations of Ni_3_(Ti, Mo) phases (A_3_B type) occur. These precipitations are formed by clustering and ordering atomic species. It is important to note that the size and distribution of precipitations are dependent on the ageing time and temperature. Whilst until the appearance of the Ni_3_(Ti, Mo) phase a minute time is needed, the Fe_2_Mo and ω precipitations arise after a much longer period. Fe_2_Mo is mostly formed at over ageing temperatures— see Figure 2. Fe_2_Mo and ω phases are considered to be coherent with the matrix. At a temperature above 400 °C, an S-phase with a hexagonal crystal structure starts to precipitate. It should be noted that the S-phase is metastable and transforms, eventually, into the ordered ω-phase.

It is also worth mentioning that 18 wt.% Ni maraging steels possess a tough martensitic structure with a high austenite reversion temperature. This austenite reversion temperature depends on the Ni content after the ageing treatment. Additionally, Maraging 350 steel has in its composition both Ti and Co in a substantial amount. Ti leads to a larger volume fraction of the Ni_3_Ti precipitations, whereas, Co facilitates the formation of the Fe_2_Mo phase. Ni_3_(Ti, Mo) phases affect the initial strength of maraging steels, whilst the Fe_2_Mo phase is responsible for the peak strength and also for sustaining high strength during prolonged ageing. The larger amount of Ni consumed in Ni3Ti precipitates reduces the Ni content in the matrix, making the high-Ni reverted austenite difficult to nucleate [25]. On the other side, Fe is consumed during the Fe_2_Mo phase formation leading to the depletion of the matrix in favour of Ni. These two precipitate formations lead to opposite effects on the reverted austenite nucleation during ageing treatment [26]. According to the TTT curves shown in Figure 2, it is possible to control the formation of reverted austenite via strict control of the ageing treatment as precipitation of Fe_2_Mo takes more time.

The abovementioned processes lead to the formation and dissolution of various precipitates and may cause changes in the thermophysical properties of the material. There is no doubt that these precipitations affect the mechanical properties of maraging steel, such as the strength and hardness of the material and even its resistance to corrosion [14]. 

### 2.4. Sample Preparation

The specimens for the thermal diffusivity test were cylindrical with a diameter of *d* = 12.65 mm and a thickness of *l* = 2.00 mm, which were cut off from a piece of metal ingot using a water-cooled cutting disc. The surface of the samples was coated with a thin layer (2–3 μm) of graphite (GRAPHIT 33 Kontakt Chemie, Zele, Belgium) to ensure high absorption of the pulse generated by a xenon flash lamp or laser flash. The density of the materials at room temperature was determined by double weighing (in air and water) using the SARTORIUS MSA125P-1CE-DA analytical balance (readability [d]: 0.01 mg). The density of the hot-work tool steel was equal to 7.75 g·cm^−3^, while for the Maraging 350 steel samples, it was 8.05 g·cm^−3^.

Samples for DSC investigations had the shape of a cylinder with a diameter *d* = 6.0 mm and they were placed in the platinum crucible with the platinum lid (volume of Pt crucible: 85 µL). The weight of the X37CrMoV5-1 hot-work tool steel sample was 219.130 mg, while in the case of Maraging 350 steel, it was 236.25 mg.

The samples for the DIL tests had a cylindrical shape with a length of 26.1 mm and a diameter of 6.0 mm for hot-work tool steel, while for Maraging 350, they were 25.4 mm and 6.0 mm, respectively. The samples for the DIL tests were cut from the bar using a water-cooled cutting disc.

### 2.5. Microstructure Analysis and Vickers Micro-Hardness Measurements

Microstructural analysis was made by using a digital microscope Keyence VHX-6000 (LM, KEYENCE Int., Mechelen, Belgium) and scanning electron microscope Phenom Pro-X (ThermoFisher Scientific, Eindhoven, The Netherlands) with an acceleration voltage of 15 kV equipped with an energy dispersive spectroscopy (EDS) chemical composition analyser. Before the microscopic observations, samples were properly polished with different grades of SiC papers and cloth polishes with additions of liquid diamond (9 µm, 3 µm and 1 µm). Final polishing was made by using an aqueous solution of silica (0.25 µm). Samples were etched with 2% Nital reagent (2% HNO_3_ + Ethanol). Hardness was measured using Vickers hardness tester Qness Q10 A+ Micro Hardness Tester (ATM Qness GmbH, part of Verder Scientific, Maastricht, The Netherlands) with a load of 10 kgf for 15 s. The mean value was calculated from at least ten measurements for every sample.

### 2.6. Thermal Analysis

#### 2.6.1. LFA

Measurements of thermal diffusivity of the tested materials were carried out independently using HyperFlash LFA 467 light flash apparatus and LFA 427 laser flash apparatus (both of NETZSCH, Selb, Germany) for comparison purposes. When the front surface of a flat-parallel specimen absorbs a short-lived (~600 ns) heat pulse generated by either a xenon lamp (LFA 467) or a laser (LFA 427), heat diffusion into the material occurs, causing an excess temperature at the back surface of the specimen which is measured by IR (CdHgTe) detector. The thermal diffusivity is calculated using a standard Cape–Lehman model with pulse correction by fitting the theoretical curve to the measurement points using the nonlinear regression method. The standard model takes into account the heat losses by radiation from the surfaces of the test sample. Thermal diffusivity tests were conducted in the temperature range (−)50 °C–480 °C using LFA 467 and independently in the RT–1000 °C range using LFA 427. Tests utilising LFA 427 and LFA 467 were performed for the first heating using argon as an inert gas with 50 mL·min^−1^ flow rate. To average the measurement results for a given temperature, two shots were generated each time. Since the LFA 467 allows the measurement of thermal diffusivity for several samples simultaneously, in contrast to the LFA 427, it is also possible to determine the specific heat and thermal conductivity of the X37CrMoV5-1 and Maraging 350 samples under test. For this purpose, it is necessary to select an appropriate reference material with similar thermal diffusivity characteristics such as Inconel 600. The specific heat capacity can be determined by the comparative method [27] by applying the following Formula (1):

(1) $$c_{p}^{s}\left(T\right)=\frac{T_{\infty }^{ref}}{T_{\infty }^{s}}\cdot \frac{\rho^{ref}}{\rho^{s}}\cdot \frac{d^{ref}}{d^{s}}\frac{Q^{s}}{Q^{ref}}\cdot \frac{V^{s}}{V^{ref}}\cdot \frac{d_{Orifice}^{2,\;s}}{d_{Orifice}^{2,\;ref}}c_{p}^{ref}\left(T\right)$$

where *d* is diameter, *V* stands for signal amplitude gain, *T_∞_* is the corrected signal of the detector voltage taking into account heat loss and it is proportional to the adiabatic temperature increase, *ρ* denotes density, *Q* is pulse energy and *c_p_* stands for specific heat under constant pressure; the superscripts *s*-refers to sample and *ref* means reference material; and the subscript *Orifice* denotes the diameter of the IR detector measuring area. Taking into account Equation (1) and the thermal diffusivity values of the tested sample *a(T)* calculated from LFA 467, the thermal conductivity $k^{s}\left(T\right)$ was calculated using Formula (2):

(2) $$k^{s}\left(T\right)=\frac{\rho_{0}}{{\left[1+\varepsilon \left(T\right)\right]}^{3}}\cdot a\left(T\right)\cdot c_{p}^{s}\left(T\right),$$

where 𝜀(𝑇) stands for the relative elongation of the sample (thermal expansion).

#### 2.6.2. DIL

Measurements of thermal expansion of X37CrMoV5-1 hot-work tool steel and Maraging 350 steel were carried out using the NETZSCH DIL 402 C (NETZSCH-Gerätebau GmbH, Selb, Germany) pushrod dilatometer in the range of RT up to about 1000 °C and the NETZSCH DIL 402 Expedis in the range of −50 °C up to about 500 °C. Nitrogen was applied as the inert gas for the DIL 402 C and helium for the DIL 402 Expedis. In both devices the flow rate of 60 mL·min^−1^ was used. The thermal expansion of the sample expressed by the coefficient of linear thermal expansion (*CLTE*) is in practice given concerning the initial length of the sample $L\left(T_{0}\right)$ − CLTE* which is also called by the NETZSCH as physical alpha (α*) given by the Formula (3) [27]:

(3) $$CLTE^{*}\left(T\right)=\frac{1}{L\left(T_{0}\right)}\cdot \frac{dL\left(T\right)}{dT}=\frac{1}{L_{0}}\cdot \frac{dL\left(T\right)}{dT}$$

The heating/cooling rate (HR/CR) was 2 K·min^−1^.

#### 2.6.3. DSC

The temperature characteristics of the specific heat capacity were determined using a differential scanning calorimeter DSC 404 F1 Pegasus (NETZSCH, Selb, Germany) in the range of RT–1000 °C. The values of specific heat were calculated using the C_p_-ratio method based on the 3-DSC curves (baseline, sapphire line and tested sample line). The test was conducted in a protective atmosphere of argon with 20 mL·min^−1^ flow rate and the heating/cooling rate (HR/CR) was 10 K·min^−1^. To obtain stable DSC signals, two evacuations of argon filling the furnace chamber were used along with 15 min isothermal segments after each completed heating/cooling cycle.

## 3. Results and Discussion

### 3.1. Microstructural Analysis

#### 3.1.1. X37CrMoV5-1 Hot-Work Tool Steel

Having its place in the class of 5% chromium hot-work steels, AISI H11 (X37CrMoV5-1) possesses extraordinary toughness and hardness, and therefore, is widely used for various applications, e.g., die steel in hot-working forging and extrusion or fabrication of helicopter rotor blades. Due to the high concentration of carbide-forming alloying elements, AISI H11 exhibits improved high-temperature softening resistance. One of the most important properties that tool steel should have is good resistance to thermal fatigue as tools used for hot working are subjected to high temperatures, and usually also to severe thermal cycling.

**Figure 3:**
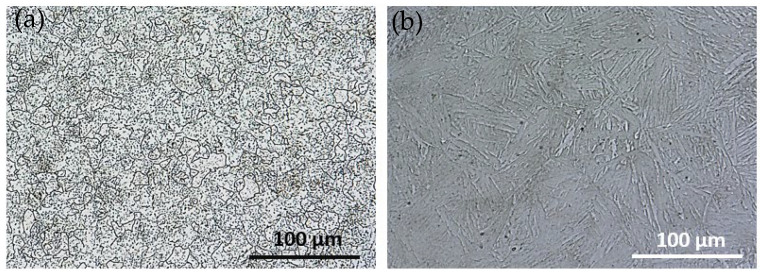
Image of X37CrMoV5-1 in (**a**) as-delivered state, (**b**) after DSC testing made by using a digital microscope.

It can be seen that the initial microstructure in the soft annealed condition consists of a ferritic (highly decomposed martensite) matrix and spherical carbides, Figure 3a). Neither carbide clusters nor remaining as-solidified carbide networks were found.

**Figure 4:**
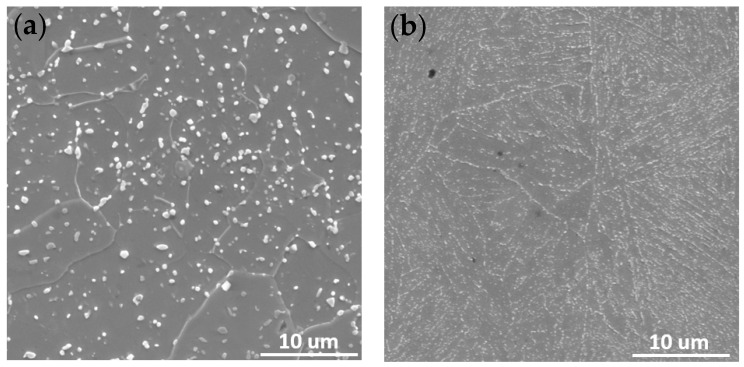
SEM image of X37CrMoV5-1 in (**a**) as-delivered state, (**b**) after DSC testing.

**Figure 5:**
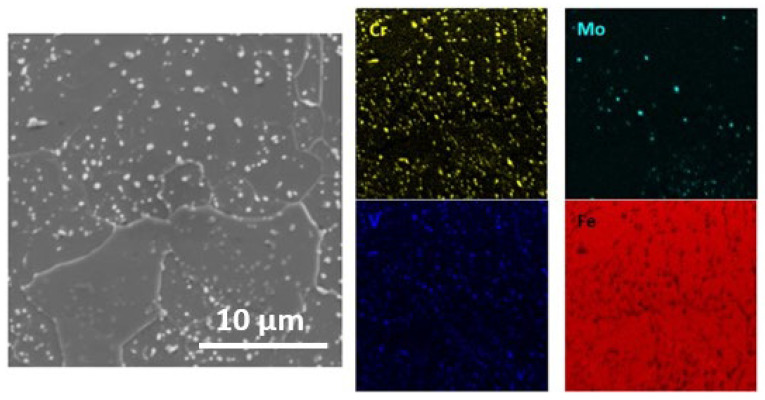
Qualitative EDS of X37CrMoV5-1 in the as-delivered state.

As shown in the SEM image in Figure 4, white particles are heterogeneously dispersed within the matrix for hot-work tool steel in the as-delivered state. Furthermore, the shape and size of the precipitates vary from round and small to irregular and big. Since this steel is composed of transition metals such as Mo, Cr, V and Mn in different contents (Table 1), they correspond to carbide precipitations. Previously, it was claimed that annealed hot-work tool steel consists of a ferrite matrix with the metal carbides such as MC (V-mainly), M_2_C (Mo-mainly), M_3_C (Fe-mainly), M_7_C_3_ (Cr-mainly) and M_23_C_6_ (Cr, Fe mainly) on the matrix when cooling from austenite [28,29]. The EDS maps exhibit how those carbides differ in chemical composition, see Figure 5.

There are significant differences in the microstructure of X37CrMoV5-1 in the as-delivered state and after DSC testing, see Figure 4. Small precipitates decorate the grain boundaries as well as are present inside grains. Precipitates observed after DSC testing in X37CrMoV5-1 are significantly smaller than those occurring in the initial condition. Moreover, laths of martensite are visible after DSC, especially in images obtained using a digital microscope, see Figure 3.

Vickers hardness HV1 measurements were 190 ± 5 and 250 ± 7 for X37CrMoV5-1 in the as-delivered state and after DSC measurement, respectively. Even though at the beginning, hot-work steel was precipitation-hardening with the microstructure composed of the ferrite matrix with spheroidal carbides, an increase in hardness was observed. This increase in hardness, compared to the hardness of the initial condition, is due to the formation of martensite together with minute precipitates that are uniformly distributed in the material.

#### 3.1.2. Maraging M350 Steel

Maraging steels are commonly used in the aerospace industry mainly thanks to their excellent combination of high tensile strength and high fracture toughness, while at the same time maintaining a relatively low weight. It is worth noting that most high-strength steels have low toughness—and the higher their strength the lower their toughness. Maraging steels owe their outstanding properties to chemical composition (low content of carbon while a large amount of nickel, cobalt, molybdenum, titanium and aluminium) as well as the manufacturing process. They are produced by the austenitising process followed by fairly slow cooling in air to form martensite. In contrast to plain carbon steel where martensite is hard, in martensitic steel, martensite is rather soft. Therefore to increase strength after quenching, maraging steel is subjected to thermal ageing during which precipitates such as Ni_3_Mo, Ni_3_Ti, Ni_3_Al and Fe_2_Mo arise.

**Figure 6:**
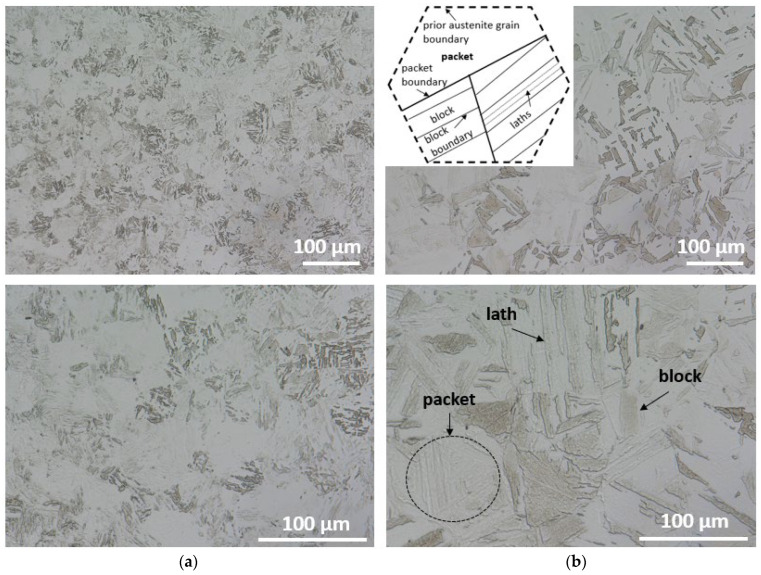
Image of Maraging 350 steel in (**a**) as-delivered state, (**b**) after DSC testing made by using a digital microscope (together with a schematic representation of lath martensite structures, e.g., laths, blocks, packets, within a prior austenite grain).

Optical micrographs of Maraging 350 samples in the initial (as-received) state exhibited uniform and equiaxed microstructure of prior austenite grains, without abnormal grain growth (Figure 6a,b). Inside prior-austenite grains martensitic microstructure with the morphology of laths, blocks and packets of the laths are observed, which is typical for this class of steels. Prior-austenite boundaries were difficult to etch in both cases. After the DSC measurements, grain growth was noticeable. Moreover, Maraging 350 steel exhibits an aged lathy martensite microstructure and the boundaries of blocks can be identified. Meanwhile, some blocks have been merged because of the growth of martensite sub-grains. The observed microstructure reveals the elongated laths with discontinuous distribution together with the increase of the spacing between them.

A slight drop in hardness was observed after the DSC test. For the sample in an as-delivered state, HV1 was 369 ± 3 whereas, for the sample after DSC measurement, HV1 equals 354 ± 3. This decrease is caused by the growth of martensite as well as the dissolution of hardening precipitates in the matrix.

### 3.2. Thermal Properties Investigations

The specific heat capacity, thermal diffusivity and thermal expansion of the X37CrMoV5-1 hot-work tool steel and Maraging 350 steel were tested in the temperature range from −50 °C to 1000 °C. Measurements of thermal diffusivity of the tested steels allowed for identifying changes in thermophysical properties quickly after the first heating run. In the case of the specific heat and the thermal expansion, the tests were repeated twice to eliminate the thermal history of the material.

Thermal diffusivity measurements were divided into two stages. First, measurements were made in the temperature range RT–1000 °C using the LFA 427 device. Temperature characteristics of the thermal diffusivity were obtained during the heating of the tested samples. In the second stage, thermal diffusivity measurements were made in the temperature range from −50 °C to 480 °C with the use of the LFA 467 apparatus. As before, thermal diffusivity tests were conducted while the test samples were being heated.

Thermal expansion measurements were carried out in two stages. In the first stage, a DIL 402C high-temperature dilatometer was used to determine the temperature characteristics of the thermal expansion of the tested steels in the RT–1000 °C range during heating at 2 K/min. In the second stage, thermal expansion and CLTE measurements were made in the temperature range of −50–500 °C using the DIL 402 Expedis also during heating and at the same rate of 2 K/min. Test results using both dilatometers were compared with each other. Two measuring cycles were carried out for each sample.

In the case of specific heat investigations, two DSC measurement cycles were carried out for each sample of both steels. In addition, a comparative method of measuring thermal diffusivity LFA 467 in the temperature range −50–500 °C was used together with the apparent specific heat measurements DSC in the temperature range RT–1000 °C, for specific heat calculations.

#### 3.2.1. X37CrMoV5-1 Hot-Work Tool Steel

**Figure 7:**
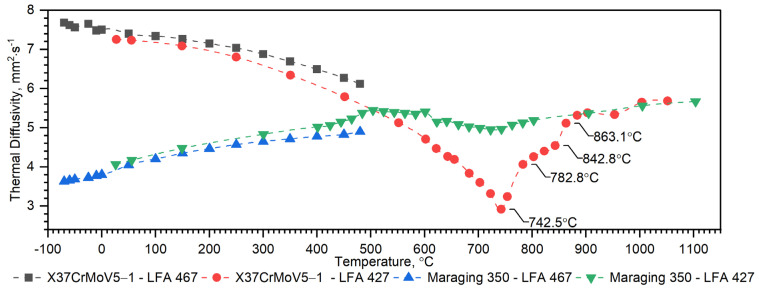
Thermal diffusivity as a function of temperature for the X37CrMoV5 −1 hot-work tool steel and Maraging 350 steel obtained from the first heating runs on LFA 467 and LFA 427.

Temperature characteristics of thermal diffusivity for the X37CrMoV5-1 samples are shown in Figure 7. In the case of X37CrMoV5-1 hot-work tool steel, the ferrite–austenite transformation occurs at a temperature of about 742.5 °C.

**Figure 8:**
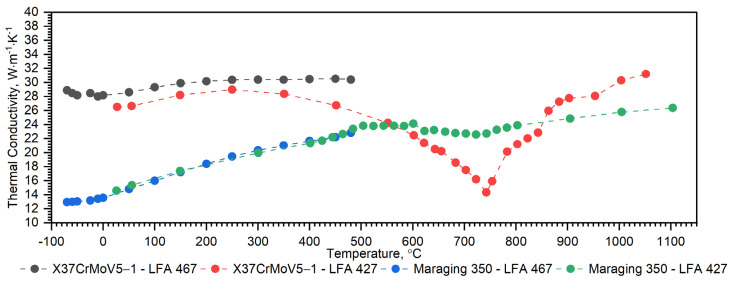
Thermal conductivity as a function of temperature for the X37CrMoV5 −1 hot-work tool steel and Maraging 350 steel obtained from the first heating runs on LFA 467 and LFA 427.

**Figure 9:**
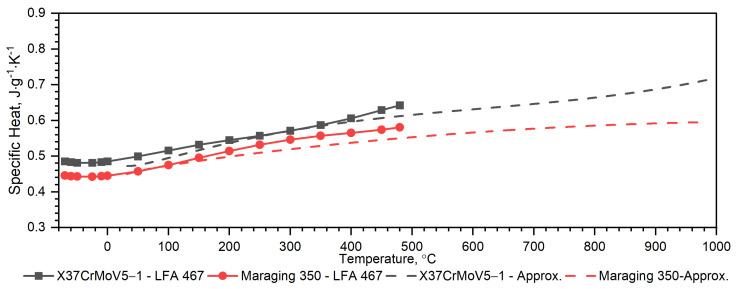
Specific heat as a function of temperature for the X37CrMoV5 −1 hot-work tool steel and Maraging 350 steel obtained from the first heating run on LFA 467 in the range from −50 °C to 500 °C and from the approximation of the experimental data on DSC in the range from RT to 1000 °C.

Figure 8 shows, for the X37CrMoV5-1 samples, the dependence of thermal conductivity as a function of the temperature obtained by the comparative method in the temperature range from −50 °C to 500 °C and using the expression (2) in the temperature range RT to 1000 °C. According to expression (2), the thermal conductivity, *k*, was calculated as a product of density, thermal diffusivity and specific heat. Figure 9 shows the dependence of specific heat as a function of temperature obtained based on DSC tests for these steels and obtained by the comparative method using the LFA 467 device.

**Figure 10:**
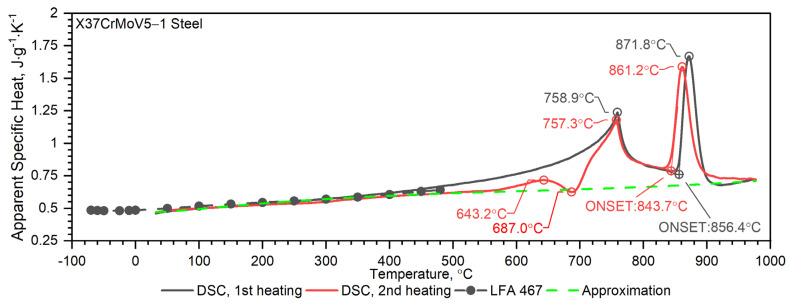
Temperature characteristics of apparent specific heat for the X37CrMoV5−1 hot-work tool steel obtained from the first and second heating run: dashed black line—results obtained from LFA 467; dashed green line—approximation of measurement on DSC.

**Figure 11:**
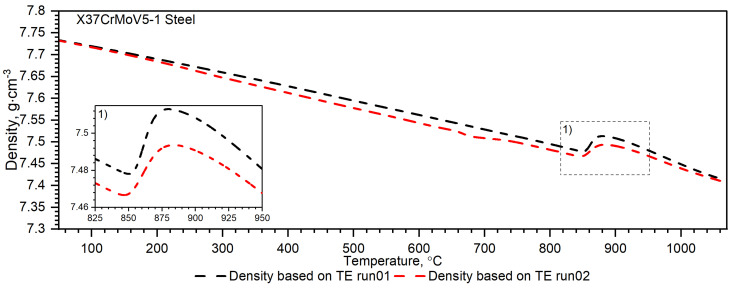
Density as a function of temperature for the X37CrMoV5−1 hot-work tool steel obtained from the first and second heating runs on DIL 402 C.

As can be seen from the results presented in Figure 7, the thermal diffusivity of X37CrMoV5-1 steadily decreases with the temperature beyond the ferrite–austenite phase transition and reaches a minimum value of about 742.5 °C. Above this temperature, thermal diffusivity as a function of temperature for this steel shows an increasing tendency. Figure 8 shows the thermal conductivity obtained by the comparative method using the LFA 467 device and calculated in the RT–1000 °C range from the measured results as a product of thermal diffusivity (obtained with the LFA 427 device), specific heat (obtained with the DSC device—Figure 9 and Figure 10) and density (obtained with the DIL 402 C device—Figure 11). Differences between the characteristics of thermal conductivity of the hot-work steel X37CrMoV5-1 in the range −50–500 °C are caused by the low accuracy of specific heat determination.

**Figure 12:**
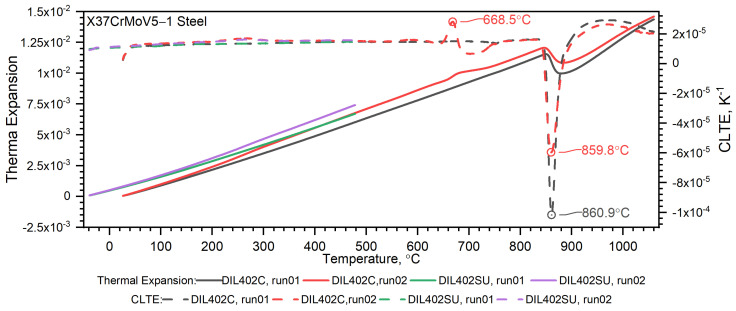
Thermal expansion dL/L_o_ (T) and CLTE as a function of temperature for the X37CrMoV5 −1 hot-work tool steel obtained from the first and second heating runs on DIL 402 Expedis and DIL 402 C.

Temperature characteristics of thermal expansion for X37CrMoV5-1 samples are shown in Figure 12.

The measurement of thermal expansion confirmed the presence of the ferrite–austenite phase transition and the shrinkage of the X37CrMoV5−1 hot-work tool steel at a temperature of about 860 °C, see Figure 11 and Figure 12. Such a high temperature of phase transition and material shrinkage, makes this steel especially predestined for the production of barrels for small arms and cannon weapons. A visible peak appears at about 680 °C in the second run and is related to the heat treatment applied to the sample after the first run, see Figure 12. Throughout the measurement range, the density decreases linearly until material shrinkage occurs, which is around 860 °C. Above this temperature, the density decreases linearly, as before.

The results of specific heat investigations for the X37CrMoV5-1 samples are shown in Figure 10. The figure also shows the specific heat calculated by the comparative method of measuring thermal diffusivity using the LFA 467 device in the temperature range −50–500 °C with the results of apparent specific heat measurements in the temperature range RT–1000 °C, obtained from DSC measurements. In the figure, the dashed line shows the specific heat as a function of temperature, described by Equation (4), which was used for the calculations according to the expression (2) of thermal conductivity as a function of temperature (Figure 8).

For the measured X37CrMoV5-1 steel sample, a correlation formula was proposed in the tested temperature range from −50 °C to 1000 °C. Figure 10 shows the fitting curve (dotted green line) for the specific heat capacity of X37CrMoV5-1 sample. The proposed formula for the X37CrMoV5-1 hot-work tool steel has the following form:

(4) $$c_{p}\left(T\left[K\right]\right)=a_{0}+a_{1}T+a_{2}T^{2}+a_{3}T^{3}+a_{4}T^{-\frac{1}{3}\;}\;\left[{J\;g}^{-1}K^{-1}\right]$$

The values of coefficients *a_i_* are given in Table 3.

The first heating DSC curve related to the X37CrMoV5-1 hot-work tool steel reveals the presence of two peaks. The first endothermic peak at 758.9 °C corresponds to the ferromagnetic to paramagnetic transformation at the Curie point, whereas the second one (871.8 °C) is linked with the transformation into γ-Fe [30,31]. If the DSC test had been conducted at even higher temperatures it would likely have yielded broad and numerous sets of endothermic peaks related to the dissolution of different carbide species [32]. In the case of the second heating, an additional broad and exothermic peak associated with carbide precipitation is observed on the DSC curve.

**Figure 13:**
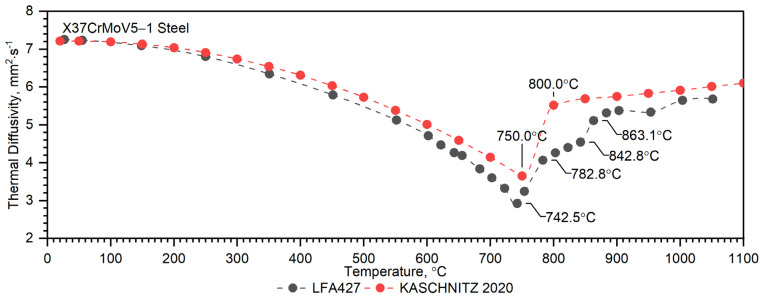
Thermal diffusivity as a function of temperature for the X37CrMoV5−1 steel obtained from the first heating runs on LFA 427 vs. literature data [15].

**Figure 14:**
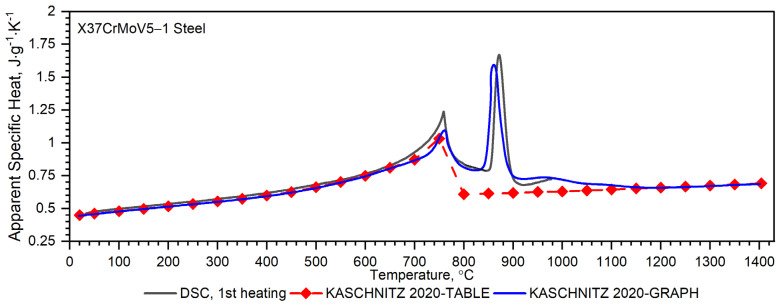
Temperature characteristics of apparent specific heat for the X37CrMoV5−1 steel obtained from the first heating run vs. literature data: dashed red line—approximation of data from [15].

A separate problem connected with DSC measurements is the method of calculating the thermophysical properties of barrel steels in order to obtain input data for the simulations of heat transfer in the barrel wall. The results of own experimental studies on the thermophysical properties of X37CrMoV5-1 sheets of steel were compared with the same studies presented in [15]. In the case of thermal diffusivity, the results are similar in the temperature range up to about 740 °C, at which temperature the ferrite–austenite phase transition occurs, see Figure 13. In the range of 740–900 °C, there are discrepancies resulting from the insufficient number of measuring points used in the work [15]. Meanwhile, this range is linked with the transformation into γ-Fe, see Figure 13 (black dotted line). In the remaining temperature range, i.e., up to about 1100 °C, the results of the thermal diffusivity are similar. In the case of apparent specific heat, the results are the same, see Figure 14. Another problem is how to correctly calculate the thermal characteristics of thermal conductivity and specific heat as inputs to the numerical simulation of heat transfer problems. Thermal diffusivity *a*, thermal conductivity *k*, specific heat *c_p_* and density *ρ* are related to the expression *a* = *k*/(*ρ*·*c_p_*). Each of these thermophysical parameters can be determined on separate measuring setups or, for example, the thermal conductivity can be calculated from the expression *k = a·ρ·c_p_*. The phase transformation is visible in each thermophysical parameter. Thus, when calculating the thermal conductivity *k* in the phase transition region from formula *k = a·ρ·c_p_*, this effect is taken into account both in thermal diffusivity and in specific heat. This means that the phase change effect and the associated enthalpy are taken into account twice [33]; this is exactly what is happening in [15]. The method of approximation of specific heat as a function of temperature, which in this case was adopted for the calculation of thermal conductivity, is shown in Figure 14. It should be noted that the approximation of specific heat did not include the peak at approx. 870 °C, because—according to the authors [22]—this was not reflected in the thermal diffusivity measurements.

#### 3.2.2. Maraging M350 Steel

**Figure 15:**
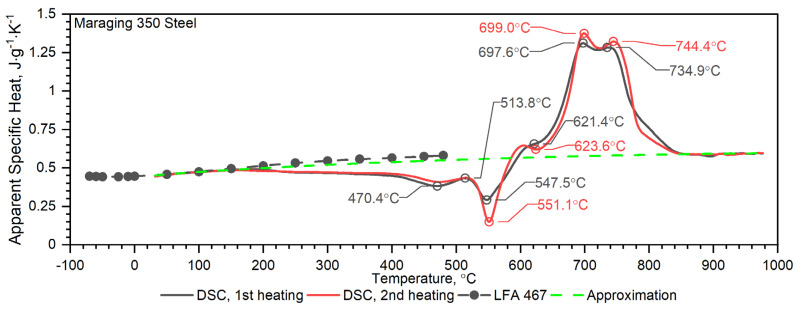
Temperature characteristics of apparent specific heat for the Maraging 350 steel obtained from the first and second heating run: dashed black line—results obtained from LFA 467; dashed green line—approximation of measurement on DSC.

**Figure 16:**
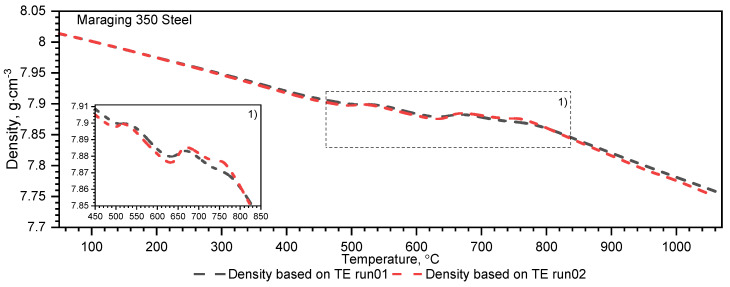
Density as a function of temperature for the Maraging 350 steel obtained from the first and second heating runs on DIL 402 C.

Temperature characteristics of thermal diffusivity for Maraging 350 samples are shown in Figure 7. Figure 8 shows the dependence of thermal conductivity as a function of the temperature for Maraging 350 samples obtained by the comparative method in the temperature range from −50 °C to 500 °C and by expression (2) in the temperature range from RT to 1000 °C. According to expression (2) the thermal conductivity, *k*, was calculated as a product of density, thermal diffusivity and specific heat. Figure 9 shows the dependence of specific heat as a function of temperature obtained based on DSC tests for these steels and obtained by the comparative method using the LFA 467 device. For the Maraging 350 steel in the entire temperature range, thermal diffusivity increases quasi-linear from 3.5 mm^2^/s to 5.5 mm^2^/s. Figure 8 shows the thermal conductivity obtained by the comparative method using the LFA 467 device and calculated in the range RT–1000 °C from the measured results as a product of thermal diffusivity (obtained with the LFA 427 device), specific heat (obtained with the DSC device—Figure 9 and Figure 15) and density (obtained with the DIL 402 C device—Figure 16).

**Figure 17:**
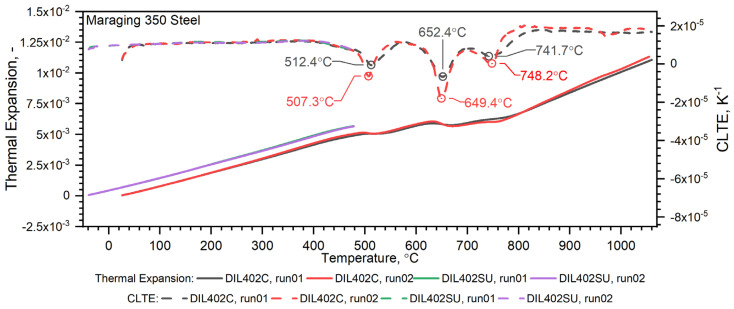
Thermal expansion and CLTE as a function of temperature for the Maraging steel obtained from the first and second heating runs on DIL 402 Expedis and DIL 402C.

Temperature characteristics of thermal expansion for Maraging 350 samples are shown in Figure 17. The density of the Maraging 350 steel drops as a function of temperature, except in the temperature range from 450 °C to 850 °C, where there is marginal shrinkage of material occurs—Figure 16.

The results of specific heat investigations for the Maraging 350 samples are shown in Figure 15. The figures also show the specific heat calculated by the comparative method of measuring thermal diffusivity using LFA 467 in the temperature range −50–500 °C with the results of apparent specific heat measurements in the temperature range RT–1000 °C, obtained from DSC measurements. In the figure, the dashed line shows the specific heat as a function of temperature, described by Equation (5), which was used for the calculations according to expression (2) of thermal conductivity as a function of temperature (Figure 8). In the case of the Maraging 350, the specific heat measurements by the comparative method also showed how to correctly approximate the DSC measurements of the apparent specific heat in the temperature range up to 1000 °C.

For the measured steel sample of Maraging 350, the correlation formula was proposed within the investigated temperature range, −50 °C to 1000 °C. Figure 15 shows the fitting curve (dotted green line) for the specific heat capacity of the Maraging 350 sample. The proposed formula for the Maraging steel has the following form:

(5) $$c_{p}\left(T\left[K\right]\right)=a_{0}+a_{1}T+a_{2}T^{2}+a_{3}T^{-\frac{1}{3}\;}\;\left[{J\;g}^{-1}K^{-1}\right]$$

The values of coefficients *a_i_* are given in Table 4.

The obtained DSC characteristic, related to a phase transition, indicates both the phase transformation and formation of precipitates in Maraging 350 steel during heating. The DSC curve for the Maraging 350 sample in the as-delivered state exhibits at least four distinct peaks. The primary exothermic peak (470.4 °C) corresponds to the recovery of martensite as well as the formation of carbides and coherent zones. These processes contributed only limited hardening effects. The second exothermic peak (about 550 °C) is in turn related to the formation of the main strengthening intermetallic precipitates such as Ni_3_(Ti, Mo) phases [16]. In the high-temperature section, two endothermic peaks can be found, one at 697.6 °C and the second at 734.9 °C, see Figure 15. The first endothermic peak is thought to be caused by the austenite reversion and formation of retained austenite by diffusion (it should be remembered that retained austenite is the austenite not transformed after cooling—the part of reverted austenite formed during heating that retains the austenite structure during the following cooling to room temperature; the other part of reverted austenite will transform back to martensite). The second endothermic peak corresponds to the transformation of martensite (HZ-hexagonal unit cell) to austenite (FCC-face cubic centred) by shear and to the resolution of precipitates or recrystallisation.

#### 3.2.3. Comparison of Thermophysical Properties of X37CrMoV5-1, Maraging 350, 38HMJ, 30HN2MFA and Duplex Steels

**Figure 18:**
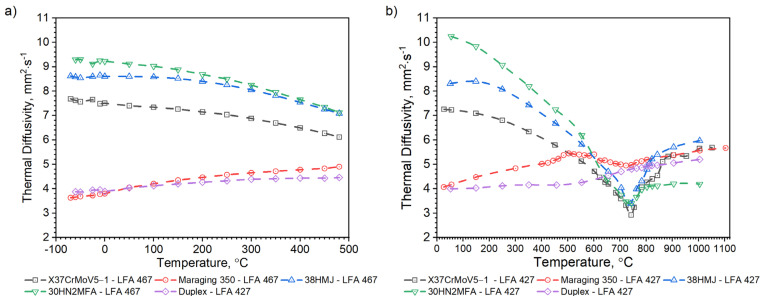
Comparison of thermal diffusivity measurements as a function of temperature for X37CrMoV5−1, Maraging 350, 38HMJ, 30HN2MFA and Duplex 2205 sheets of steel: (**a**) obtained by the authors from LFA 467; (**b**) obtained by the authors from LFA 467 [1,13].

**Figure 19:**
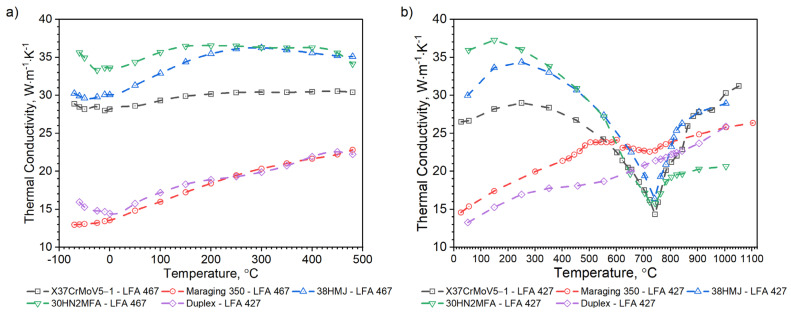
Comparison of thermal conductivity characteristics as a function of temperature for X37CrMoV5−1, Maraging 350, 38HMJ, 30HN2MFA and Duplex 2205 sheets of steel: (**a**) in the range −50–500 °C; (**b**) in the range RT–1000 °C [1,13].

**Figure 20:**
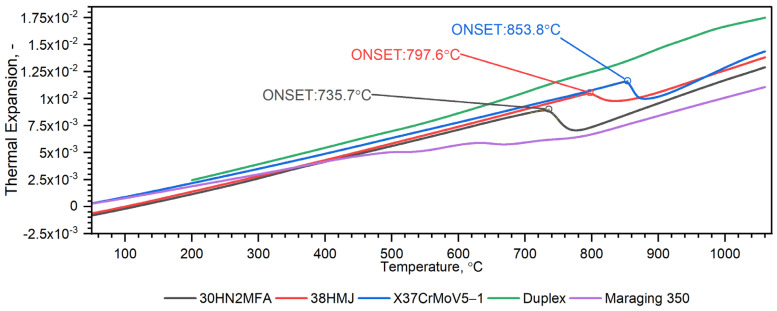
Comparison of thermal expansion characteristics as a function of temperature for X37CrMoV5−1, Maraging 350, 38HMJ, 30HN2MFA and Duplex 2205 sheets of steel [1,13].

**Figure 21:**
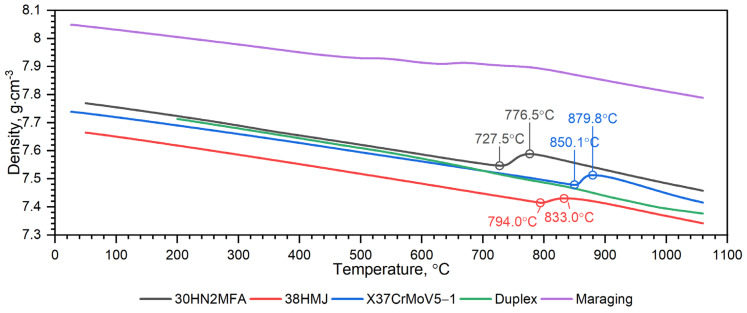
Comparison of density characteristics as a function of temperature for X37CrMoV5−1, Maraging 350, 38HMJ, 30HN2MFA and Duplex 2205 sheets of steel [1,13].

**Figure 22:**
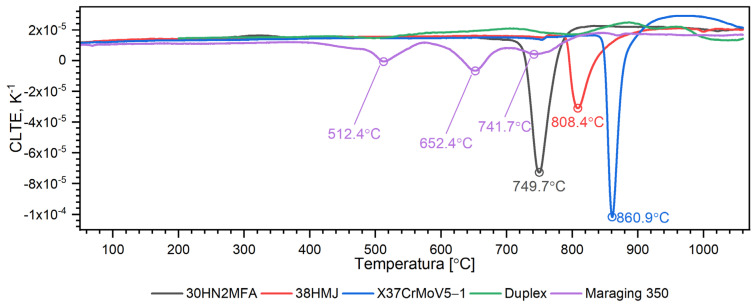
Comparison of CLTE as a function of temperature for X37CrMoV5−1, Maraging 350, 38HMJ, 30HN2MFA and Duplex 2205 sheets of steel [1,13].

**Figure 23:**
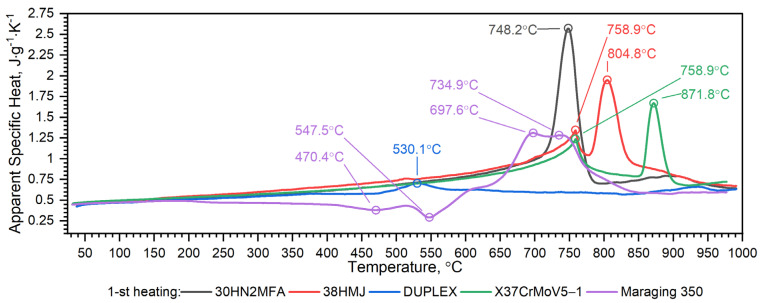
Comparison of apparent specific heat as a function of temperature for X37CrMoV5−1, Maraging 350, 38HMJ, 30HN2MFA and Duplex 2205 sheets of steel [1,13].

Figure 18, Figure 19, Figure 20, Figure 21, Figure 22 and Figure 23 show the thermophysical properties, i.e., thermal diffusivity, thermal conductivity, thermal expansion and apparent specific heat of selected barrel steels, i.e., X37CrMoV5-1, Maraging 350, 38HMJ, 30HN2MFA and Duplex, which were the subjects of the authors’ research in this paper and [1,4,13]. The comparison of the thermophysical properties of these steels in terms of suitability for the barrels of small arms and cannons consists mainly of the analysis of the shrinkage effect of the material that occurs in three of them. Thermal effects in these steels, which may affect the erosion of the inner surface of the barrel during a series of shots, are also disclosed. The overriding goal, however, was to obtain the temperature-dependent thermophysical properties of these steels as input data for numerical simulations of heat transfer in the barrel of a 35 mm calibre gun [7].

## 4. Conclusions

The authors analysed the thermophysical properties of five barrel steels commissioned by armaments factories. In terms of suitability for barrels, we can divide them into two groups, i.e., steels in which there is a ferrite–austenite phase transition and steels in which this effect does not occur. The first group includes steels 30HN2MFA, 38HMJ and X37CrMoV5-1, with a medium carbon content; the second group Duplex 2205 and Maraging 350 have a low carbon content. Only for steels from the first group, the number and order of shots are important when testing the maximum temperature of the barrel’s service life. Thermal expansion tests of steel from this group show that 30HN2MFA steel shrinks at 735.7 °C (ONSET in thermal expansion), 38HMJ steel at 797.6 °C and X37CrMoV5-1 steel at 853.8 °C. In the case of 30HN2MFA and Duplex steel, the authors tested the transient heat transfer in the 35 mm barrel wall for the sequence of 60 shots [7]. In the same way, the thermophysical properties of X37CrMoV5-1 steel and Maraging 350 steel presented in this paper should be entered as input data for calculations.

The nature of changes in thermophysical properties as a function of temperature of steel from the first group is similar. The thermal diffusivity and thermal conductivity of these steels decrease continuously with the temperature outside of the ferrite–austenite phase transition region and reach a minimum value at about 742.5 °C for the X37CrMoV5-1 steel, 743.3 °C for 30HN2 and 741.0 °C for the 38HMJ. The temperature dependence of both parameters above the minimum shows an upward trend. However, the increase in these parameters as a function of temperature is small. The results of the apparent specific heat tests of these steels revealed the existence of two peaks. The first endothermic peak at 758.9 °C for X37CrMoV5-1 and 38HMJ steels and at 748.2 °C for 30HN2MFA corresponds to the ferromagnetic to paramagnetic transformation at the Curie point. The second at 871.8 °C for X37CrMoV5-1 and at 804.8 °C for 38HMJ is related to the transformation into γ-Fe. For 30HN2MFA steel, the Curie point temperature and the γ-Fe transformation temperature probably coincide. As input to the numerical simulation of heat transfer in the barrel wall, we use only the specific heat in the form of correlation formulas.

For the second group of steels, although they differ in the method of production, the nature of changes in thermophysical properties as a function of temperature is similar. The thermal diffusivity and thermal conductivity of these steels increase quasi-linearly over the entire temperature range from about 4mm^2^/s to about 5 mm^2^/s for both steels. The results of the apparent specific heat tests of these steels revealed the existence of small peaks. In the case of Duplex 2205 steel, at a temperature of about 530.1 °C, the chromium-rich ferrite, i.e., the α’ phase, dissolves and a peak appears [13]. For Maraging 350 steel, at least four peaks appear. The nature of these peaks is extensively explained in this paper. In numerical simulations, we use only specific heat in the form of correlation formulas presented in the paper.

A separate problem raised in this paper is the method of calculating the thermophysical properties of barrel steels in order to obtain input data for the simulations of heat transfer in the barrel wall. The authors argue with the results of calculations of thermophysical properties presented in [15]. As a rule, we consider the phase transition effect only in thermal conductivity characteristics. Sometimes this effect is taken into account twice, both in thermal conductivity and in specific heat. This way of calculating these parameters should be considered incorrect.

## Figures and Tables

**Table 1:** Chemical composition of the hot-work tool (X37CrMoV5-1) steel [15].

Component	C	Mn	Cr	Ni	Mo	V	P	Si	S
Concentration [wt.%]	0.39	0.36	5.52	0.40	1.30	0.45	0.012	0.84	<0.002
	**Fe**								
	Balance								

**Table 2:** Chemical composition of the Maraging 350 steel [21].

Component	C	Si	Mn	Ni	Co	Mo	Ti	Al	Fe
Concentration [wt.%]	0.03 max	0.10 max	0.10 max	18.50	12.00	4.80	1.40	0.10	Balance

**Table 3:** Coefficients for calculating specific heat capacity of X37CrMoV5-1 samples in Equation (4).

Coefficient	Value	Coefficient	Value
$a_{0}\;\left[{J\;g}^{-1}K^{-1}\right]$	$3.7942\times 10^{-1}$	$a_{3}\;\left[{J\;g}^{-1}K^{-4}\right]$	$5.2378\times 10^{-10}$
$a_{1}\;\left[J\;g^{-1}K^{-2}\right]$	$7.7825\times 10^{-4}$	$a_{4}\;\left[{J\;g}^{-1}K^{-\frac{4}{3}}\right]$	$2.1759\times 10^{-1}$
$a_{2}\;\left[J\;g^{-1}K^{-3}\right]$	$-9.8434\times 10^{-7}$		

**Table 4:** Coefficients for calculating specific heat capacity of Maraging 350 samples in Equation (5).

Coefficient	Value	Coefficient	Value
$a_{0}\;\left[{J\;g}^{-1}K^{-1}\right]$	$4.6660\times 10^{-1}$	$a_{2}\;\left[{J\;g}^{-1}K^{-3}\right]$	$-1.1046\times 10^{-7}$
$a_{1}\;\left[{J\;g}^{-1}K^{-2}\right]$	$2.4713\times 10^{-4}$	$a_{3}\;\left[{J\;g}^{-1}K^{-\frac{4}{3}}\right]$	$-7.7045\times 10^{-2}$

## Data Availability

The results of the research conducted as part of an internal university grant were not published as a report. This publication is the only place where our test results are presented.
